# Shock and Nervous Influence in Parturition

**Published:** 1885-08

**Authors:** Henry P. Newman

**Affiliations:** Detroit Medical College. 182 North Halsted Street; Chicago


he speaks under the head of nerve-exhaustion and shock, briefly but eloquently acknowledging the poverty of literature upon the subject and appealing for a more thorough consideration of its value and import.

"Twenty years ago," he says, "these two pathological states played a conspicuous part in the etiology of sudden death in child-birth. Now the fashion has changed. Such terms as 'Nervous Apoplexy' or 'Idiopathic Asphyxia' belong to an almost forgetten nomenclature. And yet, none the less, the necessity remains to account for a class of cases in which death takes place without recognizable organic lesion."

While the writer has not suffered the misfortune of cases of marked and sudden fatality, he cannot but associate many instances of distressing and protracted after-symptoms attendant upon the puerperal state, with an abnormal nervous condition, rather than any direct local lesion.

And it is to these constantly recurring examples, rather than to the phenomenal and more scattered instances of rapidly fatal termination, that we must look for more promising and satisfactory data.

With the limited time at command, and the paucity of technical literature upon a subject pertaining as much to the domain of psychology as to any material science, we submit the following three cases in practice, as additional testimony to the importance of recognizing the different phases of nerve-influence and shock in parturition.

CASE I.—On the 5th of the present month, I was called about midnight to see Mrs. H., a healthy woman of thirty-five years, mother of five children.

I found her suffering the cutting, grinding pains of the first stage of labour, which had been in progress since early evening. The cervix was dilated to the size of a half-dollar, the membranes were intact, and the soft parts were well bathed with the usual mucous secretions.

With each paroxysm of pain, the membranes became tense, the uterus contracted moderately well and everything gave promise of the normal progress of labour.

Though apparently of strong constitution and well able to endure her share of physical suffering, I observed that the patient was greatly agitated and apprehensive, each succeeding pain having the visible effect of fretting her beyond reason, and lessening her powers of endurance.

This led me to inquire more particularly into her former history, disclosing the fact that, with the exception of the first, her previous labours, though attended by no noteworthy accident, had each been prolonged from three to five days, greatly exhausting the parturient and retarding convalescence.

The conduct of her first labour had been very unfortunate. Large and repeated doses of ergot had been exhibited, which resulted in impaction and finally forceps delivery, without anæsthesia, and badly lacerated cervix and perineum.

From the shock of such an experience the woman recovered with a very natural horror of the function of reproduction.

In the last instance, a firmly-rooted apprehension of disaster, amounting to superstition, and enhanced by the prophetic statement of her former accoucheur, that she would never live through another pregnancy, had taken complete possession of her mind.

With such a history before me, and seeing that little appreciable progress had been made in the intervening hour, I carefully examined the presenting part,—to find a possible cause of delay,—the pelvic cavity, the abdomen, vagina, bladder and rectum, with negative results, excepting that the uterus was now gradually retracting upon its contents, as evidenced by the hardening of the uterine walls and rigidity of the cervix; further, the pain was now constant and the paroxysms more severe.

Realizing that the woman was becoming needlessly exhausted, I administered a full dose of morphine hypodermically. The effect of the opiate was a quieting of the extreme reflex irritability and great relief to the sufferer.

Living at some distance from my patient, I remained at the house until the morning. The opiate was repeated at daylight and again about six A. M., when I left, with instructions to call me when pains returned with any degree of regularity or severity.

There was no summons until six o'clock in the evening. The patient was then in much the same condition as on the previous night, and she had suffered from more or less irregular contractions during the entire day.

I determined on active measures to relieve the woman and sent for an assistant with instruments. Meanwhile, an attempt was made by the fingers alone at forcible dilation of cervix. Two hours of patient and persistent effort—using chloroform to allay pain and nervous irritability,—were rewarded by sufficient dilatation to allow the introduction of Bartlett's modified Tarnier forceps. Having ruptured the membranes, the head was grasped at the superior strait. Alternate traction and relaxation simulating nature's effort, served to complete dilation at the end of another half hour, when delivery was accomplished without further trouble.

After forcible expulsion of placenta, Credé's method, the uterus contracted fairly under a single dose of ergot. The patient re-acted well, and she is at present progressing favorably, notwithstanding the unavoidable presence in the same set of apartments of two cases of scarlatina, developed on the ninth day, and a third on the twelfth, of the puerperal state.

CASE II.—On a Sunday in December, 1883, I was hastily summoned to the bedside of Mrs. D., a frail, nervous woman of middle life, a multipara. I found the patient pale and nerveless with cold extremities, feeble, thready pulse, and perspiration standing upon the forehead.

The following circumstances were related by the husband and midwife in attendance:

Labour had been in every respect normal and had proceeded as far as the second stage, when in the absence of the husband and momentary absence of the midwife, the patient had been violently startled and labour instantly arrested, by the entrance of a sneak-thief who roughly threatened her life with drawn revolver.

Examination showed the cervix fully dilated, with the head engaged in the left occipito-anterior position.

Under restorative measures, the woman rallied somewhat from her stupor, but the uterine inertia continued and I applied forceps and effected delivery without further delay.

Credé's method and ergot were employed to expel the placenta and to insure retraction of uterus.

The patient remained in the state of partial stupor, induced by the shock, throughout the night. In the morning the temperature rose to 102° F., and so continued, with but slight variation, during the ensuing four days.

On the morning of the fourth day, the temperature was 104° F., and a very mild attack of septicæmia ensued, without recognizable local inflammation. Its duration was about one week. Finally she made a slow but uninterrupted recovery. There were four cases of scarlatina in the house at the time, three of them fatal, one in the patient's family, and nursed by her until her confinement; a circumstance which may account for the septicæmia.

CASE III.—Mrs. L., primipara, twenty-one years of age, of slight and delicate appearance, but strong, "wiry," nervous constitution, was taken in labour July 11th, 1883.

The duration of the entire labour was about eight hours; the pains of the first stage were very severe and protracted, but bravely borne.

From the rupture of the membranes, which occured prematurely, until full dilatation ushered in the second stage, the suffering rapidly increased, reaching supreme agony in the violent and uncontrollable uterine contractions, attending the stage of expulsion.

Except in the somewhat tedious process of dilatation, and the suffering induced by the exaggerated reflex nervous influence in the second stage, this was in every way a normal labour.

With the culmination of anguish and the entrance of the child into the world, the mother lost consciousness.

This was superseded by great exhaustion and pallor; nausea and benumbing of the intellectual faculties, with arrest of uterine action, compelling artificial delivery of placenta.

The shock was all but fatal, and the nervous exhaustion so complete that reaction was barely induced by the use of restoratives and stimulants.

Five or six weeks of great mental depression followed, characterized by extreme indifference to child, friends and life.

During this time she suffered from pelvic peritonitis of a severe type for one uncomplicated by sepsis.

Convalescence was very slow.

Returning to Case I, I regard it as a pointed example of the inhibitory influence of the uterine nerves of cerebro-spinal origin, interfering with the rhythmical uterine action and thereby prolonging the labour.

Such a condition demands prompt interference in the first stage, to guard against the depressing influence of severe and protracted suffering, and the consequent dangers of exhaustion.

From the history of this case and the very thorough examination I made, I cannot entertain any theory that will account for the wearisome delay in delivery, except that of the disturbing influence of the woman's overpowering dread and nervous excitement.

The remembrance of the ordeal of her first childbirth, refreshed by frequent pregnancies, and the gossip of cronies, had engendered in her mind a morbid apprehension of the event, intensified by each period of suffering and the slow and fatiguing recoveries.

I am confident that had she been allowed to exhaust herself in the last instance, by prolonged and fruitless travail, the outcome would have been serious, taking into account the domestic situation which followed.

But of these three cases, No. II is perhaps the most striking and undeniable proof of the ascendency of mind over matter; a mental emotion of great violence actually preventing the completion of a physiological process.

In a less marked degree, I believe that the mental impressions which obtain during childbirth, strong in proportion to the intelligence and susceptibility of the individual, are etiological factors, which must be recognized in the handling of the puerperium.

The subsequent history of Case III has been a most flattering confirmation of these views.

She has been under my direct supervision during another pregnancy, and the value of the prophylactic treatment employed throughout the period, has been beyond estimate.

Having gained the confidence and coöperation of the patient, she was withdrawn as far as possible from the influences and associations which formerly governed her life.

The active intellectual exercises and artistic pursuits for which she had great talent, were prohibited, and the calm routine of domestic occupations substituted; absence from the city, during the heated term, supplied a grateful change of scene and surroundings.

Finally the mind was kept from dwelling upon the dreaded ordeal of labour by the assurance that she should be spared by anæsthesia the consciousness of an unnecessary degree of suffering.

Carrying a very large child, the latter weeks of gestation were attended by great discomfort, and the advent of labour was a welcome event.

BARUCH, in an article entitled, "Management of the Third Stage of Labour," comparing the labours of the refined city-bred woman and her antitype, the healty aborigine, makes the point that while in the latter the first stage is vigorously performed, and she seems to be ushered at once into the next, in the former the chronological relation between the two stages is reversed; owing to the predominance in the higher type of the sympathetic system of nerves in the first stage, and the more pronounced influence of reflex action in the second.

This is precisely what occurred in the case of Mrs. L.

In her first labour, representing the exaggerated type of the nervous woman of artificial refinement, the first stage was protracted and severe, and the second rapidly concluded. While in the second delivery, after the establishment of a more equable relation between the physical and psychical organizations, and calming of the nervous excitability, the stage of dilatation was short, the second more protracted and less violent.

Chloroform was administered to partial anæsthesia just before the rupture of the membranes and its use continued until after expulsion of placenta.

The entire labour occupied about five hours, reaction took place promptly.

There was not the slightest rise in temperature above normal during the following ten days.

The mental condition of the patent was tranquil and happy, exhibiting the natural and normal culmination of the process of parturition.

In all such cases the details of prophylaxis will suggest themselves to every intelligent accoucheur, and will accord with individual requirements.

Among the most serviceable therapeutical agents in controlling reflex nervous irritability during parturition, are our list of anæsthetics, chloroform, ether, nitrous oxide, bromide of ethyl, with chloral, bromides and the opiates.

The superiority of bromide of ethyl over chloroform as an anæsthetic is urged by Dr. Montgomery, of Philadelphia, in the June number of the *American Journal of Obstetrics.*

The claims recently made for *cimicifuga racemosa,* in a paper read before this Society, by Dr. J. S. Knox, would certainly recommend its use in this class of cases as preparatory treatment.

The last two named, like every other measure which promises to facilitate labour and mitigate the extreme and unnecessary pain frequently experienced in childbed, demand fair trial.

Rich in possibilities and suggestions as is such a subject, the purpose of this paper will have been served in drawing attention to its application in the alleviating of human suffering, which in parturient women is often sufficient to dethrone reason and destroy life; and the abolition of a long train of nervous ailments and distressing reflex symptoms incident to pregnancy and calculated to exhaust and enfeeble the maternal system; thus exposing the patient to the dangers which threaten the post-partum state.

In conclusion we offer the following suggestions:

1st. That we have a higher nervous organization presiding over the process of childbirth and subjecting it to like influences and derangements which obtain in other physiological functions.

2nd. As civilization advances, the co-relation of mind and matter becomes more intimate and complex, and calls for a proportionate advance in psychological therapeutics, and the application thereof to cases of predominant mental and nervous influences.

3rd. In many cases of so-called tedious labours the irregular contractions of the first stage are the result of an exalted state of nervous irritability.

4th. Active interference is indicated in many cases of protracted labour due to nervous influence, to guard against the dangers of exhaustion and shock.

5th. Much is to be expected from judicious prophylaxis. Especially would I urge the necessity of direct professional supervision over the entire period of gestation from the earliest months.

6th. There will still remain to be combated social, moral and educational environments, which we can scarcely expect to see abolished, until the laity, as well as the profession, is better informed as to the deleterious consequences of departure from the standard of physiological perfection in the mothers of our race, and the best means of approximating that equipoise of the mental and physical organization which it is primarily the design of nature to establish.

182 North Halsted Street.

